# A dazzle of zebras: launch of *ASM Case Reports*

**DOI:** 10.1128/asmcr.00002-24

**Published:** 2024-08-01

**Authors:** Carey-Ann D. Burnham

**Affiliations:** 1 Pattern Bioscience, Austin, Texas, USA

**Keywords:** Case Report, Case Series, Peer Review

## EDITORIAL

The American Society for Microbiology (ASM) journals are some of the most prominent and well respected in the microbial sciences. This high-quality portfolio is home to a wide variety of scholarly publications, ranging from exceptional basic and clinical research to widely read and cited review journals. Together, these journals span the microbial sciences and serve as an important vehicle to communicate the latest information and advances. A missing element in this outstanding portfolio (until now) is a home for case reports relevant to the microbial sciences. Thus, *ASM Case Reports* is launching, with the first issue anticipated in January 2025.

I believe in the utility of high-impact case reports to serve as illustrative teaching and communication tools to describe new information and demonstrate why that information is important. The act of linking clinical scenarios or syndromes to specific cases makes them less abstract and serves as a conduit to facilitate learning while tangibly demonstrating the clinical significance of a specific concept. When the topic of a case report is important, and the manuscript is well done, the paper is an enduring contribution to the scientific literature. For example, the 2003 *New England Journal of Medicine* case report on the first infection with a vancomycin-resistant strain of *Staphylococcus aureus* has been cited more than 800 times and continues to be cited in 2024 (1). Case reports were used to convey initial observations of patients experiencing *Pneumocystis* pneumonia and Kaposi’s sarcoma in the early days of the HIV epidemic (2, 3), and case reports were tools used to communicate the arrival of COVID-19 in the United States (4).

I am honored and excited to have the opportunity and responsibility to serve as the founding Editor in Chief for *ASM Case Reports*. Our goal is for *ASM Case Reports* to serve individuals in a variety of work habitats in the microbial sciences, including practicing medical technologists, clinical microbiologists, infectious disease specialists, infection prevention specialists, pathologists, public health practitioners, and regulators in academia, industry, government, and healthcare. The articles will be of interest to readers at all career stages, from early-stage trainees to seasoned professionals. *ASM Case Reports* will be a home for papers from around the world, with geographically diverse authors, sharing valuable information around the globe.

Case reports are often thought of as manuscripts describing uncommon presentations of common conditions, or common presentations of uncommon conditions. While that encapsulates some of our planned scope for *ASM Case Reports*, we strive to exceed this traditional definition. We welcome case reports describing novel clinical cases, cases illustrating the utility or scope of new diagnostic methods, as well as descriptions of new therapeutics or vaccines in action. We are seeking illustrative cases from both human and veterinary medicine and from every corner of the world.

*ASM Case Reports* will also be home to full-length Case Series articles, Commentaries highlighting and discussing highly impactful or controversial cases, and an Educational Feature that will include not only the Case Report but also additional materials, including a facilitator discussion guide and self-assessment questions. It is anticipated that the materials in the Educational Feature will be used both in the classroom setting and for self-study.

I have several long-term objectives for *ASM Case Reports*, including becoming the go-to destination to publish the most impactful case reports in clinical microbiology and infectious disease, becoming widely known and respected, becoming an important educational tool used in a variety of settings and at a variety of educational levels, and attracting authors, editors, and reviewers from around the world. To achieve these objectives, we need your help! To build this journal together, we need you to write and submit your cases. We are looking forward to receiving your manuscripts sharing interesting and useful case reports. As you are building your manuscripts, think about the story you are conveying and how you can *show*, not only *tell*, the story. Use display items when constructing your manuscript. As appropriate, include images of microbial microscopic and colony morphology, histopathology, patient imaging, and physical exam findings. Create summary tables and figures comparing the utility of diagnostic methods that may be applied for a given disease state. Consider the novelty, as well as the scientific and educational value of the message you are conveying. The CARE (CAse REports) guidelines for case reports can be a helpful tool to review and consider as you are preparing your manuscript (5).

We will also need your help contributing to the peer review process for the articles submitted to *ASM Case Reports*. Maintaining high standards in publication and scientific communication relies on the peer review system to affirm and vet the published content as valid and credible. While peer review is not without burden and challenge, the constructive feedback delivered through this process can both have a positive impact on an individual paper and function as an act of service and mentoring for the authors.

The excellent ASM Journals staff are essential to the launch of *ASM Case Reports*, and I am fortunate to work with this great group. Together, we are actively working to build out the editorial team. Like a dazzle of zebras, we plan to deliver exceptional cases that enhance the scientific landscape. I look forward to reviewing your submissions.
